# Elucidation of intrinsic biosynthesis yields using ^13^C-based metabolism analysis

**DOI:** 10.1186/1475-2859-13-42

**Published:** 2014-03-19

**Authors:** Arul M Varman, Lian He, Le You, Whitney Hollinshead, Yinjie J Tang

**Affiliations:** 1 Department of Energy, Environmental and Chemical Engineering, Washington University, St. Louis, MO 63130, USA

**Keywords:** Cell maintenance, Co-metabolism, Metabolic flux analysis, P/O ratio, Yeast extract

## Abstract

This paper discusses the use of ^13^C-based metabolism analysis for the assessment of intrinsic product yields — the actual carbon contribution from a single carbon substrate to the final product via a specific biosynthesis route — in the following four cases. First, undefined nutrients (such as yeast extract) in fermentation may contribute significantly to product synthesis, which can be quantified through an isotopic dilution method. Second, product and biomass synthesis may be dependent on the co-metabolism of multiple-carbon sources. ^13^C labeling experiments can track the fate of each carbon substrate in the cell metabolism and identify which substrate plays a main role in product synthesis. Third, ^13^C labeling can validate and quantify the contribution of the engineered pathway (versus the native pathway) to the product synthesis. Fourth, the loss of catabolic energy due to cell maintenance (energy used for functions other than production of new cell components) and low P/O ratio (Phosphate/Oxygen Ratio) significantly reduces product yields. Therefore, ^13^C-metabolic flux analysis is needed to assess the influence of suboptimal energy metabolism on microbial productivity, and determine how ATP/NAD(P)H are partitioned among various cellular functions. Since product yield is a major determining factor in the commercialization of a microbial cell factory, we foresee that ^13^C-isotopic labeling experiments, even without performing extensive flux calculations, can play a valuable role in the development and verification of microbial cell factories.

## Introduction

**Figure 1:**
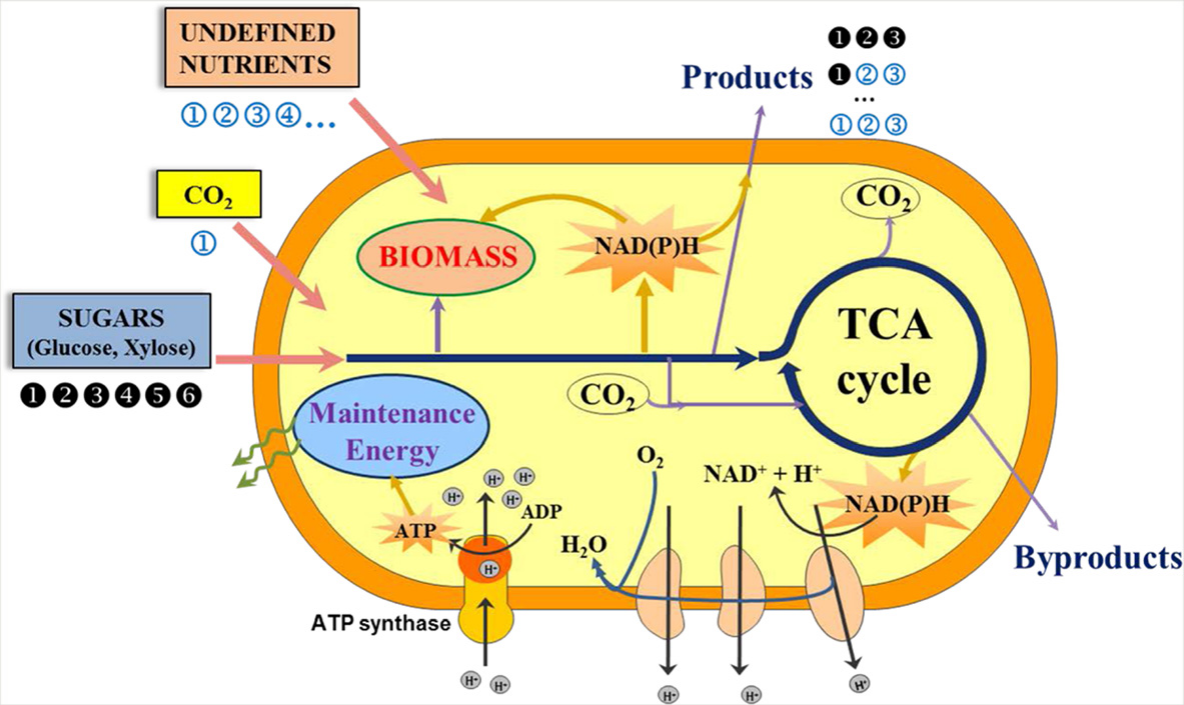
**Schematic description of microbial metabolism.** Microbes have the ability to co-metabolize diverse feedstock. Dark circles indicate labeled carbon. The enrichment of labeling in the product acts as an indicator for the relative uptake of sugars.

Recent advances in metabolic engineering have enabled us to engineer microbial cell factories for the efficient synthesis of diverse products, including bulk chemicals, pharmaceutical drugs and biofuels [1,2]. For example, advanced biofuels produced by engineered microorganisms with properties similar to that of petroleum-based fuels, have been reported extensively [3-7]. The emergence of systems biology and synthetic biology has greatly increased the potential of microbial cell factories towards the production of value-added chemicals [8-10]. For economically viable manufacture of bulk and commodity chemicals [11], the product yield is an important consideration. Researchers often include either rich medium or multiple feedstocks in microbial fermentations. Thereby, estimation of the intrinsic product yield is difficult since undefined nutrients may also contribute to the product synthesis (Figure 1). Additionally, new enzymes are often employed to improve microbial productivity [4,12-14], and the separate contributions of the heterologous and native pathways to product synthesis needs further validation. Finally, the synthesis of high-energy products (such as biofuels) requires a large amount of ATP and NAD(P)H. Due to suboptimal energy metabolism (e.g., cell maintenance cost), the actual bacterial biosynthesis is often at least three-fold lower than the amount that would be predicted from reaction stoichiometry [15].

Therefore, ^13^C-analysis is the recommended method to track the *in vivo* carbon fluxes from specific substrates to final products. Feeding microbial cultures with ^13^C-substrates results in unique isotopic patterns amongst the cell metabolites (^13^C-fingerprints) [16] to delineate metabolic pathways [17]. Integration of ^13^C-fingerprints with metabolic modeling can elucidate the intracellular metabolic fluxes (i.e., ^13^C-MFA). In the biotechnology field, ^13^C-MFA can reveal metabolic responses of microbial hosts to product synthesis and growth conditions [18-20], identify the rigid metabolic nodes that cause bottlenecks for further rational pathway engineering [21], and perform characterization of novel microbial physiologies [22-25]. In addition to these applications, ^13^C-MFA may reveal the effect of suboptimal energy metabolism on intrinsic product yields.

### Product yield using rich medium

Engineered microbes have many metabolic burdens that can inhibit both biomass growth and product synthesis. Since rich media includes both primary carbon substrates (e.g., sugars) and large amounts of nutrients (such as yeast extract), it is commonly used in fermentations to provide diverse nutrients for cell growth and stabilize the production performance of microbes [9,10]. This reduces the culture lag phase and promotes their productivity. Multiple studies have revealed that supplementing culture medium with yeast extract or terrific broth — a highly enriched medium that contains yeast extract, tryptone and glycerol as carbon sources — to engineered microbes significantly improves their final biosynthesis yields [26,27]. Since nutrient supplements can provide undefined building blocks for both biomass and product synthesis, it is difficult to precisely calculate the intrinsic product yield from rich-medium fermentation. To overcome this problem, ^13^C-analysis can gain insights into the carbon contribution from the nutrients to product biosynthesis.

**Figure 2:**
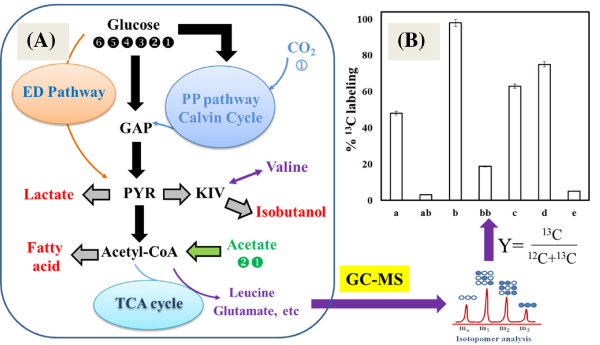
**Schematic examples to demonstrate the use of **^**13**^**C-analysis in elucidating the contributions of various carbon substrates towards the final product synthesis. (A)** Biosynthesis yield analyzed by feeding cells with ^13^C-substrates (such as fully labeled glucose and acetate). Abbreviations: GAP, Glyceraldehyde-3-phosphate; PYR, pyruvate; KIV, ketoisovalerate. **(B)** Relative product yields from a primary substrate (a – Isobutanol from glucose in a low performance strain; ab – valine from glucose in a low performance strain; b – Isobutanol from glucose in JCL260; bb – valine from glucose in JCL260) [28]; c – Free fatty acids from acetate in an *E. coli* strain [30]; d - biomass from glucose in wild type *Synechocystis* 6803 [32]; e - D-lactate from acetate in engineered *Synechocystis* 6803 [33]).

For example, two *E. coli* strains engineered for isobutanol production (i.e., a low performance strain with an Ehrlich pathway [28] and a high performance JCL260 strain with overexpression of both the keto-acid pathway and the Ehrlich pathway [29]) display an increase in isobutanol titer with the inclusion of yeast extract in their culture medium. Using fully labeled glucose and non-labeled yeast extract as carbon sources, ^13^C-experiments revealed that the low-performance strain derived ~50% of the carbons in the produced isobutanol from yeast extract (Figure 2). On the other hand, JCL260 synthesized isobutanol solely from ^13^C-glucose and used yeast extract mainly for biomass growth [28]. This observation confirms that overexpression of the keto-acid pathway overcomes bottleneck in the synthesis of isobutanol and effectively pulls the carbon flow from glucose to product. In another work, an *E. coli* strain was engineered for the conversion of acetate into free fatty acids via the overexpression of both acetyl-coA synthetase and the fatty acid pathways. During acetate fermentation, yeast extract significantly promoted fatty acid productivity, resulting in 1 g/L fatty acids from ~10 g/L acetate [30]. ^13^C-analysis of the culture with fully labeled acetate and yeast extract has shown that ~63% carbons in the free fatty acids were synthesized from ^13^C-acetate (Figure 2). Thereby, the intrinsic product yield from a primary substrate in a rich medium could be correctly estimated based on isotopomer analysis.

### Product yield during co-metabolism of multiple carbon substrates

Algal species are able to utilize both CO_2_ and organic carbon substrates. Such mixotrophic metabolisms can alleviate the dependence of algal hosts on light and CO_2_ limitations, and thus enable them to achieve high biomass growth rate and product titer [31]. ^13^C-metabolite analysis has been used to track their photomixotrophic metabolisms in different scenarios. For example, *Synechocystis* sp. PCC 6803 (blue-green algae) is capable of performing photomixotrophic growth. ^13^C-MFA has shown that CO_2_ contributes to 25% of *Synechocystis* biomass yield during its mixotrophic growth with ^13^C-glucose and ^12^CO_2_[32]. On the other hand, ^13^C-analysis has tracked D-lactate synthesis in an engineered *Synechocystis* 6803 [33]. In that study, the lactate production increased substantially during the co-metabolism of both CO_2_ and acetate. Experiments with fully labeled acetate and ^12^CO_2_ determined that nearly all of the lactate molecules were non-labeled and that only the acetyl-CoA-derived proteinogenic amino acids (leucine, glutamate and glutamine) were ^13^C-labeled. This observation suggests that acetate entered into TCA cycle and was involved only in biomass growth, while the yield of D-lactate was completely derived from CO_2_ (Figure 2). This result further indicates that acetate could inhibit the pyruvate decarboxylation reaction and thus direct more carbon flux from pyruvate to lactate. The above study shows the value of ^13^C-analysis in improving our understanding of pathway regulations for product synthesis. Since many microbial platforms (including both algal species and heterotrophs) may co-metabolize multiple carbon substrates simultaneously, isotopomer feeding can reveal the contributions of each substrate to the corresponding metabolite pools, and thus predict the potential bottlenecks in biomass or product formations.

### Product yield from alternative pathways

**Figure 3:**
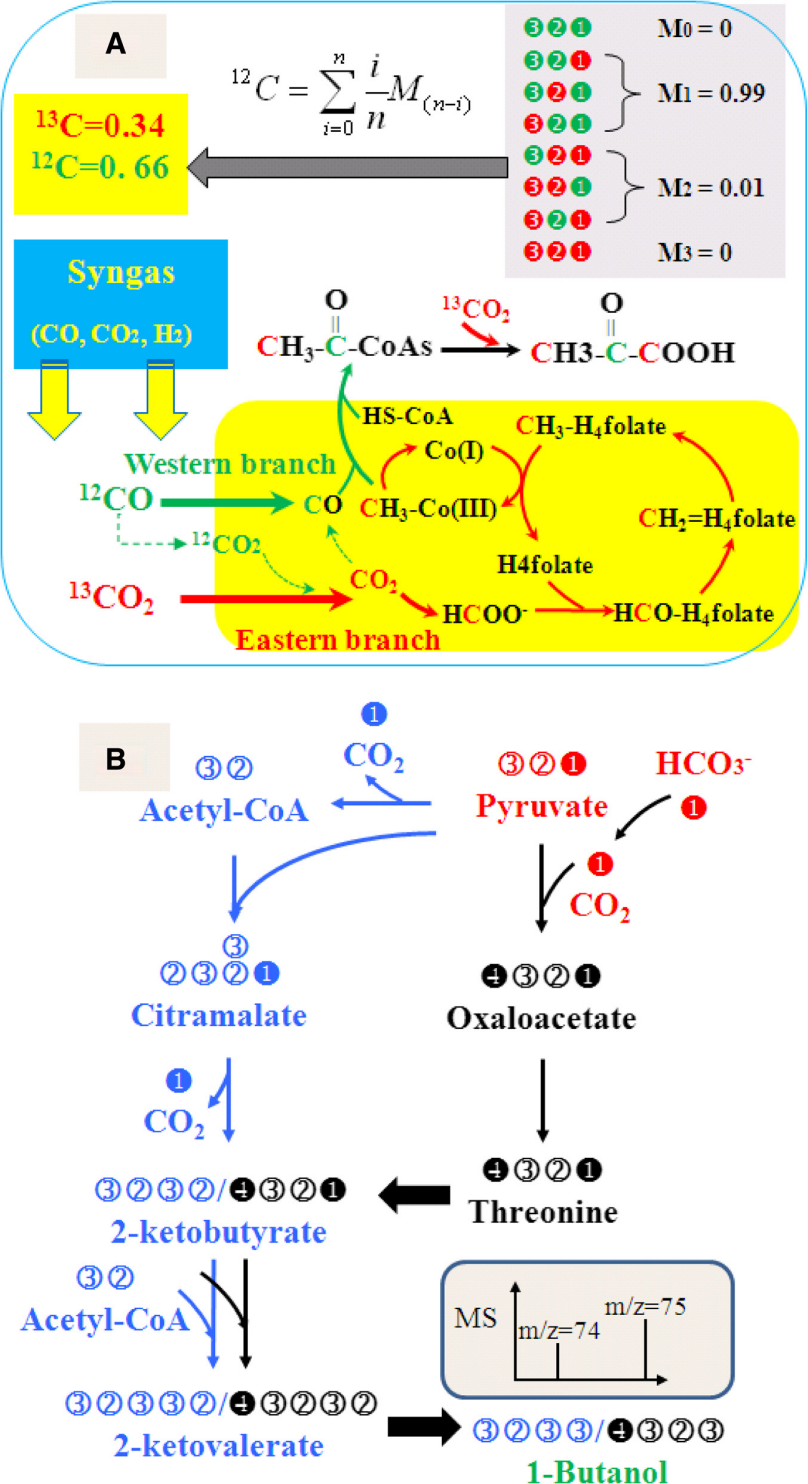
**Schematic examples illustrate that **^**13**^**C-analysis can be utilized to determine the contributions of various biosynthetic pathways towards final product yield. (A) **^13^C analysis to study the carbon assimilation during syngas fermentation (^13^CO_2_, ^12^CO and H_2_). Analysis of metabolite labeling patterns can determine CO_2_ and CO utilization for pyruvate production. The isotopomer data of pyruvate was used as a demonstration of ^13^C applications for product yield calculations. **(B)** Threonine and citramalate pathway for the synthesis of 1-butanol. The carbon rearrangement network shows the labeling of 1-butanol from the two biosynthesis pathways, when fed with 1-^13^C pyruvate and ^13^C bicarbonate.

^13^C-analysis can decipher the yield of products with multiple biosynthesis routes. For example, the acetogenic bacterium *Clostridium carboxidivorans* uses syngas (H_2_, CO and CO_2_) to generate various chemicals (e.g., acetate, ethanol, butanol, and butyrate) [34]. It contains several routes for CO_2_ fixation, which includes the Wood-Ljungdahl pathway, the anaplerotic pathways, and the pyruvate synthase reactions. ^13^C-experiments can identify the relative contribution of each CO_2_ fixation pathways towards product synthesis. As a demonstration, cultivation of *Clostridium* with labeled ^13^CO_2_ and ^12^CO has been shown in Figure 3A. Analysis of the labeling patterns in either alanine or pyruvate could reveal the relative contributions of the different CO_2_ assimilation reactions to biomass and product synthesis.

Yield of a product form a biosynthesis pathway may suffer losses from side reactions and intermediate degradation/secretion. A statistical analysis on previous metabolic engineering works observed 20% ~ 30% yield reduction per engineered enzymatic reaction step (“Rule of Thumb”) [26,27]. To reduce the carbon loss, novel pathways are constantly proposed and engineered into microbial hosts to create a “short-cut” or carbon efficient route from the feedstock to the final product. Whenever heterologous pathways are engineered into a microbe, the actual contribution to the final product of the new pathway versus the native pathway is often difficult to be estimated [35]. In the following example, we demonstrate that ^13^C-experiments can determine the relative fluxes through multiple pathways by measuring product labeling. Specifically, 1-butanol could be produced simultaneously from a threonine pathway and a citramalate pathway (a short-cut keto acid-mediated pathway) in *E. coli*[36]. If 1st position ^13^C-pyruvate and ^13^C-bicarbonate were fed to 1-butanol producing cultures, labeling patterns in 1-butanol can reveal the fluxes through both the routes (Figure 3B). Recently, a non-oxidative glycolytic cycle (NOG) was designed to increase biofuel yield [12]. This NOG pathway starts with fructose 6-phosphate and undergoes three metabolic cycles to generate acetyl-CoA without losing any carbon. To probe the contribution of NOG pathway to overall cell metabolism, this study has presented a carbon rearrangement map for ^13^C-analysis of the NOG pathway function. These examples illustrate the potential of ^13^C-analysis to examine the *in vivo* activity of various novel pathways towards product synthesis.

### Product yield influenced by bioenergetic efficiency

The theoretical product yield is generally calculated based on the stoichiometry of product synthesis from a carbon substrate. However, microbial energy metabolism also affects product yield because the synthesis of high-energy chemicals is energetically expensive, consuming large amounts of ATP/NAD(P)H. Cell maintenance (i.e., energy consumed for functions other than the production of new cell material) strongly competes for energy molecules and limits product synthesis. The maintenance energy involves regeneration of macromolecules, futile metabolic cycles, energy spilling reactions, proofreading, cell motility, preservation of chemical gradients, and repairing of cell damage caused by environmental stresses [37,38]. For example, non-growth-associated maintenance in wild type *E. coli* consumes 7.6 mmol of ATP per gram dry weight per hour [39]. Moreover, oxidative phosphorylation of NADH is a major source for ATP generation (theoretical maximum P/O ratio: 1 NADH ➔ 3 ATPs) [40]. Cytochrome oxidase is transmembrane protein complex that transfers electrons to O_2_ and translocate protons across the membrane to establish a proton gradient to power ATP synthase. However, proton translocation through membrane is not always coupled with electron transfer from NADH to O_2_, which reduces the contribution of oxidative phosphorylation to the establishment of the proton motive force for ATP synthesis [41,42]. Thereby, the actual P/O ratio, which is still in debate, is observed to be below 2.5 [43]. Under metabolic stresses, the respiration efficiency can be further reduced because trans-membrane proton gradients for ATP synthesis leak over time, resulting in loss of catabolic energy capture [37,44]. For example, the riboflavin producing *Bacillus subtilis* has a P/O ratio of 1.3, and a small increase in P/O ratio (from 1.3 to 1.5) could increase riboflavin yields by 20% [45].

**Figure 4:**
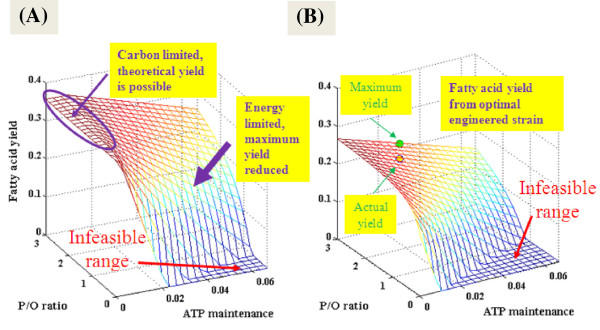
**3D illustrations of relationships among theoretical yield, P/O ratio and non-growth associated ATP maintenance. (A)** Theoretical Yield as a function of P/O ratio and non-growth associated ATP maintenance without constraining biomass growth (v(8) ≥ 0). **(B)** Theoretical Yield as a function of P/O ratio and non growth associated ATP maintenance at growth rate v(8) = 3.6. The units of yield and ATP maintenance are ‘g C16:0 fatty acid/g glucose’ and ‘mol ATP /g glucose’ respectively. Under certain circumstances, the energy cannot be balanced for fatty acid or biomass production, resulting zero yield [47].

The amount of energy from substrate catabolism diverted to non-growth functions varies dramatically depending on different organisms and growth conditions (e.g., during *E. coli* growth, its energy yield of substrate catabolism could be one-third of the theoretical maximum) [37]. To illustrate the impact of energy efficiency on product yield [46], a small-scale flux balance model related to fatty acid-overproducing strain was built exclusively for this report. This small-scale model employs eight reactions (Table 1) to demonstrates free fatty acid production as a function of non-growth associated ATP maintenance and P/O ratio [47]. The fluxes were resolved by the function below:

$$\begin{array}{l} \max\;v\left(2\right)\\ \text{such}\;\text{that}\;A\cdot v=b\;\text{and}\;\mathrm{lb}\;\leq \;v\;\leq \;\mathrm{ub}, \end{array}$$

where the objective function is to maximize v(2) (i.e., the relative flux of fatty acid). A is the reaction stoichiometry. *lb* and *ub* are upper and lower bound for each reaction flux, v(i). Figure 4A shows the relationship between maximum yield, P/O ratio and ATP maintenance without constraining biomass growth (v(8) ≥ 0) (Table 1). A higher P/O ratio makes the microbial system less sensitive to the increased demand for ATP loss. When the ATP maintenance is low and the P/O ratio is close to 3, the fatty acid yield can reach the theoretical value of 0.36 g fatty acid/g glucose (Figure 4A). In such conditions, eliminating competing pathways or engineering new pathways to avoid carbon loss may be effective to achieve a yield close to the theoretical maximum [48-50]. When ATP consumption for maintenance increases, cells need to use extra carbon substrates for energy generation, thereby decreasing the fatty acid yield significantly. Under these circumstances, one should consider strategies that will either reduce cell maintenance or increase the flux towards ATP synthesis.

**Table 1:** Simplified biochemical reactions considered in the model

**Flux, v**	**Reactions**	**Note**
v(1)	Glucose ➔ 2AceCoA + 2ATP + 4NADH	Glycolysis
v(2)	AceCoA + 1.75NADPH + 0.875ATP ➔ 0.125 C16:0 fatty acid	Fatty acid synthesis
v(3)	AceCoA ➔ 2NADH + NADPH + ATP + FADH2	TCA cycle
v(4)	NADH ➔ NADPH	Transhydrogenation
v(5)	NADH ➔ P/O ATP	Oxidative phosphorylation
v(6)	FADH2 ➔ 0.67(P/O)ATP	Oxidative phosphorylation
v(7)	ATP ➔ ATP_maintenance	ATP maintenance (non-growth associated)
v(8)	6.6Glucose + 37.6ATP + 9.5NADPH + 2.5AceCoA ➔ 39.7Biomass + 3.1NADH	Biomass formation

Note: Glucose consumption for both biomass growth and product synthesis is normalized to 100. The linear optimizer ‘linprog’ function in MATLAB is used for the optimization. The final yield (g fatty acid/g glucose) is calculated as follows: Y = (v(2)/8∙256)/(100∙180) g C16:0 fatty acid/g glucose.

In a recent study of an engineered *E. coli* for fatty acid overproduction [47], ^13^C-MFA showed that the theoretical ATP/NADPH generation (assuming P/O ratio = 3) from glucose catabolism was much higher than ATP/NADPH consumption for biomass growth and fatty acid synthesis. After optimization of biosynthesis pathway via 'push-pull-block' strategies, this engineered strain had a fatty acid yield of only 0.17 g fatty acid/g glucose (Figure 4B) because a substantive fraction of energy yield from glucose catabolism was lost due to the suboptimal energy metabolism. Such high cell maintenance and low P/O ratio in the engineered *E. coli* are likely caused by the various physiological stresses during biofuel overproduction (e.g., changed cell membrane integrity and compositions [51]). Thereby, ^13^C-MFA not only applies for a better understanding of carbon flux distribution, but also provides a diagnostic analysis of the energy-dependent metabolic capability for product yields. If the microbial metabolism demands a considerable amount of ATP/NAD(P)H for both biosynthesis and cell maintenance, optimal product yield is unlikely to be achieved by overexpressing biosynthesis pathways or by redirecting metabolic fluxes to avoid carbon losses. A more promising approach would be to improve energetic prosperity or respiration efficiency, thereby allowing the cells to “burn” substrates more efficiently to satisfy the energy requirement [52,53].

## Conclusions

Product yield is one of the main considerations of microbial cell factories [54]. Microbial productivity is not only associated with the efficiency of biosynthesis enzymes, but is also intertwined with the energy metabolism [55]. Simple ^13^C analysis can characterize the hosts’ intrinsic production yields under different carbon sources, and determine the contributions of the different pathways to biosynthesis. In addition, ^13^C-MFA can profile microbial fluxomes and determine the amount of extra substrates that the cell consumes to compensate for ATP losses from diverse cellular processes, which is essential to understand metabolic capability of a microbial host for maximal product yields. In the end, ^13^C-analysis, using the labeled product as internal standards, can also be employed to correct product measurement noises in fermentation processes due to water loss, product evaporation or degradation [56]. This review paper aims to emphasize the indispensable value of ^13^C-labeling techniques to the metabolic engineering field as we foresee an extended use of ^13^C-experiments for the development of microbial cell factories.

## Competing interests

The authors declared that they have no competing interests.

## Authors’ contributions

AMV/YJT wrote the introduction, product yield from rich medium, multiple carbon substrates and parts of yield from alternative pathways. LH wrote the bioenergetic efficiency. LY contributed to the section of product yield from alternative pathways. WH polished the paper. All the authors approved the final manuscript.
